# Supplementary material for: Knowledge, attitudes, and practices of face mask utilization and associated factors in COVID-19 pandemic among Wachemo University Students, Southern Ethiopia: A cross-sectional study
Source: PLoS One. 2021 Sep 20;16(9):e0257609. doi: 10.1371/journal.pone.0257609 (PMC8451998; doi:10.1371/journal.pone.0257609)
Supplement: Attachment — Submitted filename: response to the reviwers.docx [file pone.0257609.s002.docx]

**Response to Reviewers**

1. Is the manuscript technically sound, and do the data support the conclusions? For authors yes really b/se the manuscript must describe a technicall1y sound piece of scientific research with data that supports the conclusions. Experiments must have been conducted rigorously, with appropriate controls, replication, and sample sizes. The conclusions must be drawn appropriately based on the data presented/ findings.
2. Has the statistical analysis been performed appropriately and rigorously? Yes I agreed and I have also data in our hand and attached it as an additional supporting document and I have one concern to reviewer two b/se the response is no and I would try to attach cleaned munscrupit after a line by line correction.
3. Have the authors made all data underlying the findings in their manuscript fully available? Yes I would try to keep up PLOSE one guideline policy and I have a data availability statement in the manuscript as a section and I don’t get the idea of reviewer three, if not clear in this cleaned munscrupit after correction you welcome.
4. Is the manuscript presented in an intelligible fashion and written in Standard English? Yes, I accept which forwarded to me from the reviewers and I tried to correct it.
5. Concerns about dual publication, research ethics, or publication ethics? This research munscrupit was not summited elsewhere and the study was conducted after ethical approval was obtained from the Institutional Review Board (IRB) of the Wachemo University (WCU) College of Medicine and Health Sciences and I would try to prepare this research munscrupit as PLOSE one guideline for publication.
6. Methods section: The results of the students tested in the pilot study were included in the study? Answer no the study subjects which are included under primary study from out of the main campus mean in branch campus called Durame and the actual data should be collected in main campus regular students and for better to see inclusion and exclusion criteria in addition to data quality assurance
7. How did the authors use the dependent variable? The answer I used to measure the face mask utilization primarily practice part of the tools b/se those individuals knowing may /may not be practiced good, if the students from having practiced good face mask use we call it students have a good practice and not it poor and better to refer the operational definition part of the research articles [my primary outcome used to measure our outcome variables was face mask utilization was practiced].
8. Many questionnaire items are not suitable for the subtitled domain; hence don't reflect the targeted domain? Yes, you are right and I would try to make correct it and remove the variables with repetition.
9. The introduction? I would try to correct it see the cleaned munscrupit
10. The methods regarding how the study population was selected are unclear? I don't think it better to specify it? Specifically, if the university has approximately twice as many male students, why is the study population primarily female? You are right and say to say this and my data also similar, but by default, I will change place like sex of male and I would try to correct it. I have one arguing idea, this is always not correct, depending the data collected and it may also depend on Bach and in some university male students may be always greater than female, this idea it may /may not be always correct and no must be seen e.g. the study conducted in A Cross‑Sectional Study of Knowledge, Attitude, and Practice Towards Face Mask Use Amid the COVID‑19 Pandemic Amongst University Students in Vietnam, 2021.
11. The survey questions themselves, and how they were scored, are unclear. Were they provided to the participants exactly as shown in the appendix? How were the questions developed? Answer sees the operational deft ion part and sees the primary data on how I measured it in cleaned munscrupit.

The text of the results section repeats a lot of what is in the tables and figures, and there are typographical errors? Yes really I tried to correct all things and see the evidence(I would try to use different expert and academician and I would try to show also for free sample editors like scribendi customer services with mail {customerservice@scribendi.com},and [Grammarly](https://app.grammarly.com/) and I would try to make paraphrase online ) and if any extra I need to hear from you all.

12. Abstract: Only one set of results is presented in the abstract. Was this the primary outcome? Yes of course.
13. Lastly but no least I have tried to address the comments which is forwarded you’re your side and the munscrupit which was attached with track change is not highlighted because the whole munscrupit document should be modified for typographical errors, finally, I would try to address your comment as much as I can and I thank those all reviewers for constructive idea and suggestion and a lot of things changed and try to see the cleaned munscrupit which is attached to you. I hear good news thank you a lot!!!!!!!!!
