# Supplementary material for: Knowledge, attitudes, and practices of face mask utilization and associated factors in COVID-19 pandemic among Wachemo University Students, Southern Ethiopia: A cross-sectional study
Source: PLoS One. 2021 Sep 20;16(9):e0257609. doi: 10.1371/journal.pone.0257609 (PMC8451998; doi:10.1371/journal.pone.0257609)
Supplement: Attachment — Submitted filename: Response to reviwers .docx [file pone.0257609.s003.docx]

**Rebuttal letter**

**Academic Editor**

1. Formatting was edited as PLOS ONE's style requirements,

2. ORCID added

3. Additional details regarding participant consent was revised as

“The ethical approval was obtained from the ethical review committee (ERC) of the Wachemo University (WCU) College of Medicine and Health Sciences. Before beginning the investigation, permission was obtained from Wachemo University, colleges and departments. Participants were given written consent about the study, its goals, effects, and the significance of the data before they were enrolled. To ensure confidentiality, all information was rendered anonymous”.

4. Language usage, spelling, and grammar were checked and revised as much as possible.

5. Additional information regarding the validation and development of the questionnaire was added as a supporting file.

6. All relevant data were in the document and we provide for the journal if needed

7. Ethics statement was in the Methods section of our manuscript. Revised

8. Captions for your Supporting Information files were revised.

**Response for reviewers**

**Reviewer #1:**

**The introduction** section was minimized and tried to revise the flow of ideas and study rationale.

The sentences in lines 193, 195, 196 were corrected

**Methods** section: The results of the students tested in the pilot study (pre-test) were not included in the actual study. The pilot study (pre-test) was done in the Durame campus that is out of the main campus.

The **dependent variable** primarily uses to measure was practice. The correct response of > 14 out of 18 questions (>80%) was considered as good practice and ≤ 14 (≤80%) was considered as poor practice. The questionnaire items language revision and proofreading (revised and corrected). Many questionnaire items are suitable for the subtitled domain, hence does reflect the targeted domain and items were with repeated meanings and difficult to be understood in the questionnaire were removed and corrected.

**The results section and discussion** were revised after correcting the methods section.

**The conclusion** does reflect the results and it also based our primary significant findings and the awareness campaigns and educational session were used rather than training sessions.

**Reviewer #2:**

1. **The introduction** section was minimized and the sentences in lines 55 were corrected and we will focus on the main point of the paper.
2. The method **section** on how the study population was selected is clear and revised. The university has approximately twice as many male students as female and this not always not correct(We have one arguing idea, this is always not correct, depending on the data collected and it may also depend on Bach and in some university male students may be always greater than female, this idea it may /may not be always correct and no must be seen e.g. the study conducted in A Cross‑Sectional Study of Knowledge, Attitude, and Practice Towards Face Mask Use Amid the COVID‑19 Pandemic Amongst University Students in Vietnam, 2021) and by default, we make mistake we revised and corrected it.
3. The survey questions were scored and were clear and now attached to you as supporting information and it was developed after reviewing different kinds of literature.
4. **The results section** was not repeated in the tables and figures at similar times and typographical errors like spelling, grammar, Indentation, Font Size, Font Style, and Line Spacing.: revised and corrected see the document attached to you as like an others.
5. The manuscript is edited by a different expert see document summit in others part as ZIPD.

**Reviewer #3:**

**Abstract:** Yes of course the only primary outcome (practices) presented in the abstract.

**Introduction:** The references for the statements made in the introduction lines 85, 88, and110 were provided and corrected. We tried to minimize and revise the flow of ideas in the introduction sections in general.

For line 115: the difference between pre-asymptomatic (infected with the virus but have not yet acquired symptoms) and asymptomatic (individuals who do not have symptoms but are infected with the virus) were corrected and revised.

**Independent variables:** corrected and revised for those independent and dependant variables, see on measurement of variables munscrupit part.

**The objectives** for the primary/secondary outcomes of the study were to determine the knowledge, attitude, and practices of face mask utilization and associated factors in the COVID-19 pandemic among Wachemo University students.

The predictor’s variables like **religion, marital status** does include in the final analysis and they should be included only to know the disruptive status of the college students and these variables don't have any effect on outcome variables in general.

**Living situation**: these variables would affect the outcome? Yes really in our study(those students having a history of rented accommodation were 76.5% times less likely to have negative attitudes than on face mask utilization than those students were having living in the dorm(AOR: 0.235;95%CI: 0.093,0.590)); it might be due to lesser exposure status for covid 19 pandemic.

Discussion: we tried to add potential explanations for the difference: corrected and revised and we tried to add limitations of this study.

**Conclusion:** comprehensive training about face mask utilization should be designed and given by authorities corrected and revised as comments given us by **Reviewer #1**

The study population was young students with, high education background, so results may be generalizable to a similar study population based on our findings, so conclusions were based on our findings to a similar study population.

### The English language/grammar, with particular attention, was given to tense, punctuation, and sentence structure. Revised and tried to be corrected

6. We do not want our identity to be public for this peer review.

**For all reviewers:**

Lastly but not least we have tried to address the comments which were forwarded by your side and the munscrupit which was attached with track change was highlighted, finally, we would try to address your comment as much as we can, and in general we thank all reviewers for constructive idea and suggestion and a lot of things changed and try to see the munscrupit which is attached to you. I hear good news thank you a lot!
