# Supplementary material for: Knowledge, attitudes, and practices of face mask utilization and associated factors in COVID-19 pandemic among Wachemo University Students, Southern Ethiopia: A cross-sectional study
Source: PLoS One. 2021 Sep 20;16(9):e0257609. doi: 10.1371/journal.pone.0257609 (PMC8451998; doi:10.1371/journal.pone.0257609)
Supplement: Attachment — Submitted filename: Rebuttal Letter.docx [file pone.0257609.s004.docx]

**Rebuttal Letter**

**Responses for Editor Comments**

**Thank you for your comments!**

We’ve checked your comments and we tried to address the	issues raised:
1. we removed duplicate figures in our "FIGURE.docx" file:updated and revised.
2. We renamed the file name of our figures as Figure 1, Figure 2, Figure 3, Figure 4 and Figure 5: revised and updated.

3. We removed the duplicate cover letter.
