# Supplementary material for: Knowledge, attitudes, and practices of face mask utilization and associated factors in COVID-19 pandemic among Wachemo University Students, Southern Ethiopia: A cross-sectional study
Source: PLoS One. 2021 Sep 20;16(9):e0257609. doi: 10.1371/journal.pone.0257609 (PMC8451998; doi:10.1371/journal.pone.0257609)
Supplement: Attachment — Submitted filename: Response for reviwer.docx [file pone.0257609.s005.docx]

**Rebuttal Letter**
**Response for Editor Comments**
Thank you for your comments you give

1. The tables and figures in the order in which they appear in the munscrupit: corrected and revised.
2. All references in the Reference list were checked and corrected that they contained all relevant information: revised and updated.
3. We tried to correct grammatical and punctuation errors as much as we can: revised and updated.

**Response for Reviewers' comments:**
our figure files to the Pre-flight Analysis and Conversion Engine (PACE) digital diagnostic tool, <https://pacev2.apexcovantage.com/> based on it:
